# *Leishmania* Infection-Induced Proteolytic Processing of SIRPα in Macrophages

**DOI:** 10.3390/pathogens12040593

**Published:** 2023-04-13

**Authors:** Hana Hirai, Jing Hong, Wataru Fujii, Chizu Sanjoba, Yasuyuki Goto

**Affiliations:** 1 Laboratory of Molecular Immunology, Graduate School of Agricultural and Life Sciences, The University of Tokyo, Tokyo 113-8657, Japan; 2 Laboratory of Biomedical Science, Graduate School of Agricultural and Life Sciences, The University of Tokyo, Tokyo 113-8657, Japan

**Keywords:** visceral leishmaniasis, SIRPα, ectodomain shedding, ADAM

## Abstract

The shedding of cell surface receptors may bring synergistic outcomes through the loss of receptor-mediated cell signaling and competitive binding of the shed soluble receptor to its ligand. Thus, soluble receptors have both biological importance and diagnostic importance as biomarkers in immunological disorders. Signal regulatory protein α (SIRPα), one of the receptors responsible for the ‘don’t-eat-me’ signal, is expressed by myeloid cells where its expression and function are in part regulated by proteolytic cleavage. However, reports on soluble SIRPα as a biomarker are limited. We previously reported that mice with experimental visceral leishmaniasis (VL) manifest anemia and enhanced hemophagocytosis in the spleen accompanied with decreased SIRPα expression. Here, we report increased serum levels of soluble SIRPα in mice infected with *Leishmania donovani*, a causative agent of VL. Increased soluble SIRPα was also detected in a culture supernatant of macrophages infected with *L. donovani* in vitro, suggesting the parasite infection promotes ectodomain shedding of SIRPα on macrophages. The release of soluble SIRPα was partially inhibited by an ADAM proteinase inhibitor in both LPS stimulation and *L. donovani* infection, suggesting a shared mechanism for cleavage of SIRPα in both cases. In addition to the ectodomain shedding of SIRPα, both LPS stimulation and *L. donovani* infection induced the loss of the cytoplasmic region of SIRPα. Although the effects of these proteolytic processes or changes in SIRPα still remain unclear, these proteolytic regulations on SIRPα during *L. donovani* infection may explain hemophagocytosis and anemia induced by infection, and serum soluble SIRPα may serve as a biomarker for hemophagocytosis and anemia in VL and the other inflammatory disorders.

## 1. Introduction

Visceral leishmaniasis (VL) is the most severe form of leishmaniasis caused by the *Leishmania donovani* complex. According to the World Health Organization (as of February 2023), there are an estimated 50,000–90,000 new cases of VL annually, and the fatality rate of this disease is over 95% if left untreated. The main clinical manifestations of VL are fever, weight loss, hepatosplenomegaly, and anemia. Anemia is very common in VL patients, with an overall prevalence higher than 90% [1]. Although there are various reports on the factors associated with anemia in VL, and the cause of anemia should not be derived from single factor, one proposed cause associated with anemia is hemophagocytosis [1].

Hemophagocytosis describes the phagocytosis of hematopoietic cells by hyperactivated macrophages and occurs in 7~75% of VL patients in their bone marrow [1]. Increased hemophagocytic activity is a phenomenon observed not only in VL but also in other infectious diseases and can occur as the disease progresses [2,3]. Secondary hemophagocytic lymphohistiocytosis (HLH) is a life-threatening hyperinflammatory syndrome characterized by hemophagocytosis and occurs as a complication associated with several disorders including VL [4]. In fact, VL patients showing hemophagocytosis are often accompanied with fever, pancytopenia, and splenomegaly, which is consistent with the criteria of secondary HLH [1,5].

Signal regulatory protein α (SIRPα) is one of the receptors responsible for regulating phagocytosis and host immune activation, expressed mainly in neuronal cells and myeloid cells such as macrophages, dendritic cells, and neutrophils [6]. When a ligand binds to the extracellular domain of SIRPα, four Tyr residues in immunoreceptor tyrosine-based inhibitory motifs (ITIMs) of its intracellular domain become phosphorylated, and recruit and activate SH2 domain-containing protein tyrosine phosphatases SHP-1 or SHP-2, which send generally negative signals to cells [6]. The interaction of SIRPα and CD47 on RBCs prevents hemophagocytosis [7,8] and contributes to the maintenance of RBC homeostasis by protecting intact RBCs or clearing aged RBCs [9,10]. In fact, Ishikawa-Sekigami et al. revealed that mice that expressed mutant SIRPα lacking the cytoplasmic region had moderate anemia and showed accumulation of RBCs in the spleen [11].

In a mouse model of VL, upregulated hemophagocytosis in the spleen of infected mice was observed [12] and the expression of SIRPα on macrophages decreased during *L. donovani* infection [13], indicating that changes in inhibitory signals associated with SIRPα expression may enhance hemophagocytosis during VL. In fact, SIRPα is known to modulate the immune response of expressing cells by altering their structure and expression. In in vitro experiments, both a TLR3 agonist and a TLR4 agonist decreased *Sirpa* mRNA and canceled the suppression of SIRPα [14,15]. In addition, shedding of extracellular region of SIRPα on macrophages induced by inflammation stimulation enhanced the NF-κB pathways and STAT-1 pathway [16].

SIRPα is also known to be cleaved at its extracellular domain and produce a soluble form. The shedding of cell surface receptors is a well-known biological event and has significant roles in the regulation of inflammatory responses [17,18,19,20]. In some cases, receptor shedding brings synergistic outcomes by the loss of receptor-mediated cell signaling and competitive binding of the shed soluble receptor to its ligand. Soluble SIRPα has a variety of biological functions including presynaptic maturation [21,22], the alteration of macrophage phagocytic activity [23,24], and the regulation of cell migration [25]. Recently, there were some reports on increases in serum soluble SIRPα. Vladimirova et al. detected increased soluble SIRPα in human sera after an LPS challenge [26]. Increase serum or urinary SIRPα is also reported in chronic kidney diseases [27,28,29]. Thus, an increase in serum soluble SIRPα may reflect inflammatory conditions at local sites, and can be used as a biomarker for detecting inflammatory disorders.

In order to understand the mechanisms of SIRPα regulation in *L. donovani*-infected mice and to investigate its clinical significance, we examined whether an increase in serum levels of soluble SIRPα can be induced. In addition, we examined if the parasite infection promotes ectodomain shedding of SIRPα on macrophages in vitro. Furthermore, the effect of *L. donovani* infection on the intracellular domain of SIRPα in macrophages was also examined.

## 2. Materials and Methods

### 2.1. Mice, Cells, and Parasites

Female BALB/cA mice were purchased from Japan Clea, Tokyo. All mice were kept under specific pathogen-free conditions. The mice were used for experiments at the age of 6 weeks old. Experimental infection of mice with *L. donovani* was conducted as previously described [12]. Briefly, *L. donovani* promastigotes in the late log or stationary phase were washed and resuspended with PBS at a concentration of 1 × 10^8^ cells/mL. Mice were infected with 1 × 10^7^ *L. donovani* promastigotes by intravenous injection into the tail vein. For treatment, the infected mice were administered intravenously with a single dose of 500 μg liposomal amphotericin B (AmBisome; Dainippon Sumitomo Pharma, Osaka, Japan) at 12 weeks of infection. Mice were sacrificed at either 12 or 24 weeks of infection by cardiac puncture under isoflurane anesthesia to collect the whole blood, spleen, and liver.

Murine macrophage cell line RAW264.7 cells, originally derived from tumors induced with the Abelson leukemia virus, were purchased from ATCC (ATCC TIB-71). Cells were cultured at 37 °C and 5% CO_2_ in RPMI 1640 (Sigma, Japan or WAKO, Osaka, Japan) containing 10% heat-inactivated fetal bovine serum (HI-FBS) (Thermo Fisher Scientific, Waltham, MA, USA), 100 U/mL penicillin, and 100 µg/mL streptomycin (WAKO). The supplemented RPMI 1640, called complete RPMI 1640, was used for macrophage culture unless otherwise noted.

*L. donovani* promastigotes (MHOM/NP/03/D10, a gift from the National BioResource Project at Nagasaki University [30]) were maintained in medium 199 (Invitrogen, Waltham, MA, USA) and supplemented with 10% HI-FBS, 100 U/mL penicillin, and 100 µg/mL streptomycin at 25 °C. In some experiments, *Leishmania* promastigotes were stained with CFSE (Dojindo Laboratories, Kumamoto, Japan). *L. donovani* promastigotes were incubated with PBS containing 50 µg/mL CFSE at room temperature for 30 min, then washed with RPMI 1640 three times.

### 2.2. Measurement of Soluble SIRPα

Serum levels of soluble SIRPα in naïve or *L. donovani*-infected BALB/cA mice were measured with a Mouse SIRPα ELISA kit (RayBiotech, Peachtree Corners, GA, USA). To measure the level of soluble SIRPα in the supernatants of RAW264.7 cells, 2.5 × 10^4^ cells were seeded in 96 well plates for 4 h and either stimulated with 100 ng/mL LPS or infected with *L. donovani* (MOI: 50) for 24 h. Then, the supernatants were collected and soluble SIRPα was measured using the same ELISA kit. 

### 2.3. Western Blotting

To detect soluble SIRPα in supernatants, 7.2 × 10^5^ RAW264.7 cells were seeded in 6 well plates. After 4 h, wells were washed with serum-free RPMI 1640 and incubated with 100 ng/mL LPS (Sigma) or *L. donovani* (MOI: 50) in serum-free RPMI 1640 for 24 h. Supernatants were collected and concentrated with Amicon Ultra-0.5 with PLGC Ultracel-10 membrane (Millipore, Germany). Concentrated supernatants were mixed with an SDS sample buffer and boiled for 5 min. The macrophages were also lysed in an SDS sample buffer. The total cellular, soluble, and transmembrane proteins were separated using electrophoresis on acrylamide gel, then transferred to a polyvinylidene difluoride membrane. After blocking the polyvinylidene difluoride membrane with 4% skim milk, the membrane was probed with rat anti-SIRPα extracellular domain (SIRPα-ex) antibodies (P84, eBioscience, San Diego, CA, USA) and rabbit anti-GAPDH antibodies (GeneTex, Irvine, CA, USA), diluted with PBS containing 0.05% Tween 20 (PBS-T) plus 0.4% skim milk. After washing with PBS-T three times, it was probed with horseradish peroxidase (HRP)-linked goat anti-rat IgG antibodies (Invitrogen) or HRP-linked donkey anti-rabbit IgG antibodies (GE Healthcare, Chicago, IL, USA), diluted with PBS-T containing 0.4% skim milk. The bands were visualized using an enhanced chemiluminescence detection system (GE Healthcare) and analyzed with a LAS-3000 mini (Fujifilm, Tokyo, Japan).

To detect transmembrane SIRPα in cell lysates, cells were seeded and incubated with 100 ng/mL LPS (Sigma) or *L. donovani* (MOI: 50) in complete RPMI 1640. After 24 h, cells were washed with serum-free RPMI 1640 and processed as already described. Rabbit anti-SIRPα intracellular domain (SIRPα-in) antibodies (Raybiotech) were used for detecting the intracellular region of SIRPα.

### 2.4. ADAM10 Inhibition Assay

RAW264.7 cells (2.5 × 10^4^ cells/well) were cultured in complete RPMI 1640 in 96 well plates. The cells were pretreated with complete RPMI 1640 containing 0, 1, or 10 µM GI254023X (Sigma) for 2 h. Then, the cells were either stimulated with LPS (100 ng/mL) or infected with *L. donovani* (MOI = 50) in complete RPMI 1640 containing 0, 1, or 10 µM GI254023X. After 24 h, the supernatants were collected and soluble SIRPα was measured using a Mouse SIRPα ELISA kit.

### 2.5. Quantitative RT-PCR

RAW264.7 cells were either stimulated with 100 ng/mL LPS or infected with *L. donovani* (MOI: 50) for 24 h as described above. RNA was extracted using a TRIzol reagent (Invitrogen). The concentration of total RNA was measured using a DU730 Life Science UV/vis spectrophotometer (Beckman Coulter, Chaska, MN, USA), and 4 µg of total RNA was used as the template for the synthesis of cDNA. A tube containing 500 ng of oligo (dT)16 and 10 nmol of dNTPs (Fisher Scientific, Loughborough, UK) with template RNA was incubated for 5 min at 65 °C. Then, 5× first-strand buffer, 200 nmol of DTT (Thermo, Waltham, MA, USA), and 200 U of M-MLV (Thermo) were added, and the tube was incubated at 37 °C for 50 min. The reaction was stopped by incubation for 15 min at 70 °C. The synthesized cDNA was used for the expression analyses of *Sirpa* and *Actb*. The designed primers are listed in Table 1 [15]. A real-time polymerase chain reaction (PCR) assay was conducted using 1 µL of reverse transcription PCR product as the template and 10 µL of SYBR Select Master Mix (Thermo) with the Applied Biosystems QuantStudio 5 Real-Time PCR System (Thermo). Data were analyzed with 2−ΔΔCt methods through normalization with *Actb*. The thermal cycling conditions were 95 °C for 10 min, followed by 40 cycles at 95 °C for 15 s and 60 °C for 1 min.

### 2.6. Statistical Analysis

Statistical comparisons were performed using the one-way ANOVA followed by Dunnett’s multiple comparison test, or the two-way ANOVA followed by Tukey’s multiple comparison test with GraphPad Prism 9 software (GraphPad Software, San Diego, CA, USA). A difference between groups was considered statistically significant when the *p*-value was less than 0.05.

## 3. Results

### 3.1. Increased Serum Soluble SIRPα in L. donovani-Infected BALB/cA Mice

**Figure 1:**
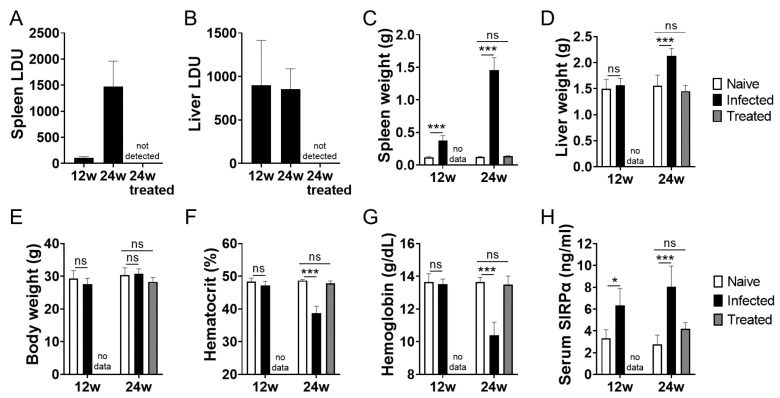
Increase serum SIRPα in *L. donovani*-infected mice. BALB/cA mice were infected with *L. donovani.* At 12 weeks of infection, some mice received treatment with AmBisome. Representative data of spleen LDU (**A**), liver LDU (**B**), spleen weight (**C**), liver weight (**D**), body weight (**E**), hematocrit (**F**), hemoglobin (**G**) and serum SIRPα (**H**) of naïve, *L. donovani*-infected, or infected/treated BALB/cA mice at 12 or 24 weeks post infection. Mean and SD of each group are shown (*n* = 5 for each group). * *p* < 0.05, *** *p* < 0.001, ns = not significant with two-way ANOVA followed by Tukey’s multiple comparison test.

*L. donovani* infection caused hepatosplenomegaly and anemia in BALB/cA mice as previously reported [12]. Parasite infection was progressive or sustained over the course of the 24-week infection in the spleen and liver (Figure 1A,B). The spleen and liver of the infected mice at 24 weeks post infection became significantly larger in size than those of uninfected mice, and the infected mice exhibited anemia represented by lower hematocrit and hemoglobin levels (Figure 1C–G). Treatment with AmBisome successfully cleared the parasites in the infected mice and led to clinical improvement represented by reduced spleen and liver sizes, as well as improved hematological parameters (Figure 1A–G). Next, serum levels of SIRPα in naïve, *L. donovani*-infected, and infected/treated mice were examined using ELISA which detect extracellular regions of SIRPα. As shown in Figure 1H, the increase in serum SIRPα levels became evident as early as 12 weeks of infection, which was before the onset of anemia. In addition, serum SIRPα levels in the treated mice were lower than those of untreated mice and were equivalent to the naïve mice (Figure 1H).

### 3.2. Ectodomain Shedding of SIRPα in Macrophages Infected with L. donovani

**Figure 2:**
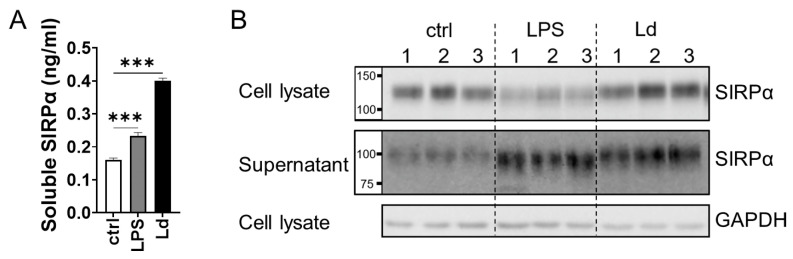
Release of soluble SIRPα from macrophages during *L. donovani* infection. (**A**) Soluble SIRPα in the supernatants was measured with sandwich ELISA. Mean and SD of the representative of two independent experiments with similar results are shown. *** *p* < 0.001 with one-way ANOVA followed by Dunnett’s multiple comparison test. (**B**) Both concentrated supernatants and cell lysates were analyzed with Western blotting using anti-SIRPα-ex antibodies. Representatives of two independent experiments with similar results are shown.

To examine whether secretion of soluble SIRPα is induced in macrophages by *L. donovani* infection, murine macrophage cell line RAW264.7 cells were treated with LPS or infected with *L. donovani* and the culture supernatants were examined for SIRPα secretion. SIRPα levels in the supernatants were higher in both treatment groups compared with those of untreated cells (Figure 2A). To investigate the molecular size of SIRPα in the supernatant, cell lysates and supernatants were analyzed with Western blotting using anti-SIRPα-ex antibodies. SIRPα detected in the supernatants had a smaller apparent molecular mass than that in the cell lysate (Figure 2B), indicating the presence of the cleaved form of SIRPα in the supernatants.

### 3.3. Involvement of ADAMs in SIRPα Ectodomain Shedding by L. donovani Infection

**Figure 3:**
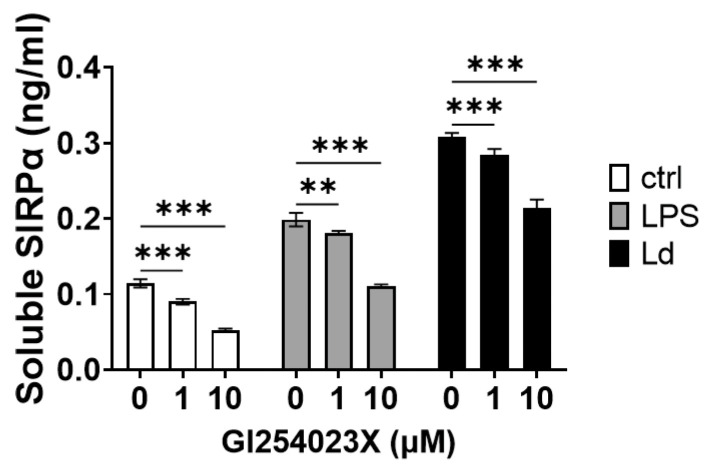
Partial inhibition of SIRPα cleavage by an ADAM10 inhibitor. RAW264.7 cells were incubated with GI254023X for 2 h and then LPS or *Leishmania* promastigotes (MOI = 50) were added to the medium (final concentrations: GI254023X = 0, 1, 10 µM, LPS = 100 ng/mL). After 24 h, supernatants were collected, and soluble SIRPα in the supernatants was measured using sandwich ELISA. Mean and SD of triplicates in each group are shown. Representative of two independent experiments with similar results. ** *p* < 0.01, *** *p* < 0.001 with two-way ANOVA followed by Tukey’s multiple comparison test.

Extracellular region of SIRPα can be cleaved by sheddases such as matrix metalloproteinase 9 (MMP-9) [25], a disintegrin and metalloprotease 10 (ADAM10) [16], and ADAM17 [31]. Since LPS stimulation induces cleavage of SIRPα by ADAM10 [16] or ADAM17 [31], the function of these ADAMs in LPS stimulation and *Leishmania* infection was compared using an inhibitor. Addition of an ADAM10 inhibitor, GI254023X, decreased the amount of soluble SIRPα in supernatants in both LPS stimulation and *L. donovani* infection (Figure 3). The degree of decrease with GI254023X treatment was less than 50% in both cases (Figure 3).

### 3.4. Loss of Cytoplasmic Portion of SIRPα during L. donovani Infection

**Figure 4:**
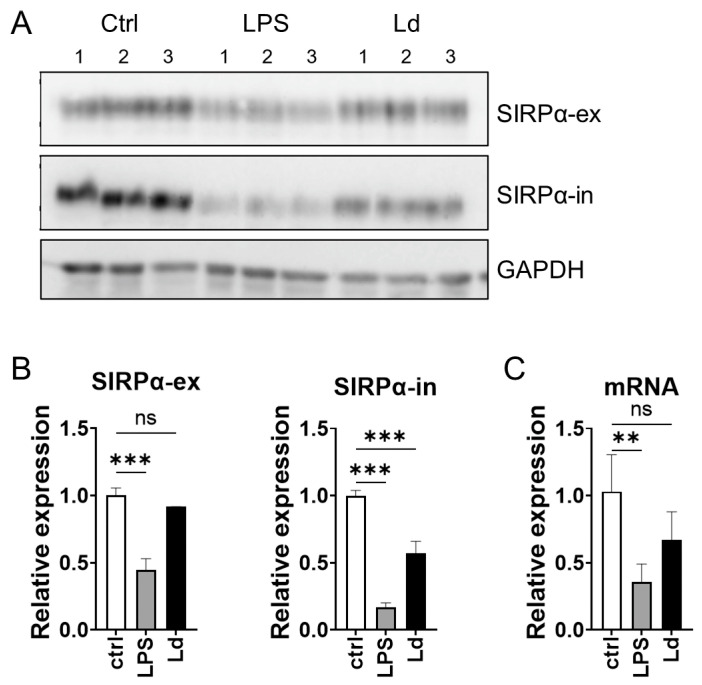
Decrease in C-terminal-competent SIRPα during LPS stimulation and *Leishmania* infection. (**A**) RAW264.7cells were incubated with medium only, 100 ng/mL LPS, or *L. donovani* promastigotes (MOI = 50) in complete RPMI1640. After 24 h, cells were harvested and the cell lysates were analyzed with Western blotting using anti-SIRPα N-terminal antibodies or anti-SIRPα C-terminal antibodies. Data shown are triplicate samples representative of two independent experiments with similar results. (**B**) Densitometric analyses with ImageJ software showing band intensity for SIRPα detected using anti SIRPα-ex or SIRPα-in antibodies. Mean and SD of triplicates in each group are shown. (**C**) Expression levels of *Sirpa* mRNA in RAW264.7 of naïve, LPS stimulation, or *L. donovani* infection were analyzed using qPCR. ** *p* < 0.01, *** *p* < 0.001, ns = not significant with one-way ANOVA followed by Dunnett’s multiple comparison test.

The increased soluble SIRPα in supernatants is possibly mediated by the cleavage of the molecule at the extracellular domain. On the other hand, it is known that the cleavage of SIRPα can occur at its intracellular domain as well [32]. Therefore, to examine the changes in the intracellular domain of SIRPα during *L. donovani* infection, antibodies against both extracellular and intracellular domains of SIRPα were used for Western blotting. When anti-extracellular domain antibody was used, a decrease in SIRPα was observed only in LPS stimulation (Figure 4A,B). On the other hand, when anti-intracellular domain antibodies were used, a decrease in SIRPα was observed in both LPS stimulation and *L. donovani* infection (Figure 4A,B). The expression levels of *Sirpa* mRNA in RAW264.7 cells declined in LPS stimulation but not in *L. donovani* infection (Figure 4C).

## 4. Discussion

In this study, structural changes of SIRPα on macrophages during *L. donovani* infection were revealed. First, we found that *L. donovani* infection induces the release of soluble SIRPα, which has a smaller molecular size than SIRPα in whole cell lysate, suggesting that *L. donovani* infection can promote shedding of SIRPα in macrophages. Regardless of the promoted shedding, no obvious decrease in SIRPα in cell lysate was observed in the infected macrophages (Figure 2). It may be because the portion of cleaved SIRPα was not large compared with the total amount. It has been reported that LPS induces ectodomain shedding of SIRPα in macrophages [16]. During the ectodomain shedding by LPS stimulation, ADAM10 cleaved the extracellular domain of SIRPα adjacent to the transmembrane domain, and then the intracellular portion of the truncated SIRPα is cleaved by γ-secretase [16]. It is difficult to elucidate from the Western blotting results whether the extracellular cleavage sites are identical between LPS stimulation and *L. donovani* infection. Nonetheless, there should be some similarity in SIRPα ectodomain shedding induced by the two different stimuli. GI254023X inhibited the release of soluble SIRPα to a similar extent during both LPS stimulation and *L. donovani* infection (Figure 3). Although GI254023X can inhibit the enzymatic activities of both ADAM10 and ADAM17, the selectivity is towards ADAM10, as shown by the IC_50_ for ADAM10 and ADAM17 which are 5.3 and 541.0 nM, respectively [33]. Together, the results in this study indicate that SIRPα cleavage induced by *L. donovani* infection is partially dependent on ADAM10, as reported for the LPS-induced cleavage [16]. On the other hand, the result that inhibition of SIRPα cleavage by GI254023X was not perfect indicates the involvement of the other sheddases in this process, including ADAM17. In the future, it is necessary to use a more specific inhibition technique for ADAM10 and ADAM17 expression, including RNAi or gene knockout, to further elucidate the detailed mechanisms of *L. donovani*-induced SIRPα cleavage.

In accordance with the in vitro result, we found that *L. donovani*-infected mice have increased serum levels of soluble SIRPα (Figure 1). It is only recently that reports on increase in serum soluble SIRPα have started to appear in the literature. Only a few papers have shown increased serum SIRPα in diseased conditions [26,27,28], and our present study is the first report of increased serum SIRPα in parasitic diseases. These suggest that, although not fully understood yet, serum SIRPα can be used as a biomarker for SIRPα shedding at inflammatory sites in the body. For instance, Shen et al. demonstrated the increase in soluble SIRPα in bronchoalveolar lavage of mice with LPS-induced acute lung injury [23]. In the case of experimental VL, the active shedding site may be the spleen, where macrophages are heavily parasitized with *L. donovani* at 24 weeks of infection [12,13]. The heavily infected splenic macrophages have a low expression level of SIRPα [13]. Together with the result in this study on ectodomain shedding of macrophage SIRPα by *L. donovani* infection (Figure 2), it is likely that increased serum SIRPα in *L. donovani*-infected mice reflects the ectodomain shedding of SIRPα in splenic macrophages. One of the notable features of soluble SIRPα is the up-regulation of phagocytosis. In cancer therapy, recombinant soluble SIRPα can bind to CD47 on tumor cells and disrupt the CD47-SIRPα interaction on macrophages, inducing phagocytosis of tumor cells [24]. In LPS-induced acute lung injury, soluble SIRPα promoted phagocytosis of apoptotic neutrophils by bone marrow-derived macrophages [23]. Therefore, soluble SIRPα may be one of the factors promoting phagocytosis of ‘self’ cells and may be involved in enhanced phagocytosis of erythrocytes in *Leishmania* infection through masking CD47 on red blood cells. In this study, we chose an MOI of 50 for infection of RAW264.7 cells with *L. donovani* in order to achieve high parasitization as a characteristic of infection-induced hemophagocytes [12,13]. The high MOI may not be suitable to observe physiological responses of macrophages to Leishmania infection. Therefore, it is necessary to address parasite dose-dependent induction of SIRPα shedding in a future study. 

It is of interest that cleavage of SIRPα induced by *L. donovani* infection was not limited to its extracellular domain. Western blotting analysis using cell lysate suggested that the loss of the extracellular domain and intracellular domain in membrane-bound SIRPα do not occur evenly during *L. donovani* infection (Figure 4). The results suggest that the observed SIRPα down-regulation is not simply derived from decreased expression of the full-length protein. In fact, *Sirpa* mRNA levels were not suppressed in *L. donovani* infection (Figure 4). Therefore, a marked decrease in SIRPα detected using anti-SIRPα-in antibodies indicates that the intracellular domain undergoes some proteolytic processing. On the other hand, the short SIRPα indicative of cleavage of the intracellular domain was not evident in the Western blotting analysis using anti-SIRPα-ex antibodies. This is possibly because the missing portion of the intracellular domain was too small to be detected with Western blotting. Since cleavage of the intracellular domain of SIRPα by serine protease in PMNs is already reported [32], it is possible that similar processing occurs on SIRPα in macrophages during *L. donovani* infection. Another possibility is that a protease derived from *Leishmania* causes cleavage of the intracellular domain of SIRPα. Lack of the intracellular portion of SIRPα can promote accumulation of RBCs at the spleen and the development of anemia in mice [11], indicating that hemophagocytosis and anemia in VL can be simulated. However, there are some reports that the removal of an inhibitory signal by a CD47–SIRPα interaction is not sufficient for prominent hemophagocytosis and that the additional activation of macrophages is needed to induce hemophagocytosis [34,35]. Therefore, although the loss of the intracellular region of SIRPα may contribute to hemophagocytosis during VL, the extent of the involvement is unclear. To determine whether the intracellular and extracellular changes of SIRPα identified in this experiment is involved in hemophagocytosis and anemia during VL, it is necessary to create cleavage-resistant SIRPα and examine the changes of pathogenesis.

In summary, *Leishmania* infection induces both extracellular and intracellular modification of SIRPα in macrophages and may contribute to hemophagocytosis and anemia. Although the involvement of SIRPα in the pathogenesis of VL needs further investigation, serum SIRPα may be used as a biomarker for the severity of VL.

## Figures and Tables

**Table 1:** List of primers for Quantitative RT-PCR.

Gene	Forward	Reverse
*Sirpa*	TCGAGTGATCAAGGGAGCAT	CCTGGACACTAGCATACTCTGAG
*Actb*	GTTACCAACTGGGACGACA	TGGCCATCTCCTGCTCGAA

## Data Availability

Data presented in this study will be available from the corresponding author upon request.
